# Early hospital readmission following stroke: the Florida Stroke Registry

**DOI:** 10.3389/fstro.2023.1238442

**Published:** 2023-08-02

**Authors:** Hannah Gardener, Jose G. Romano, Terry Derias, Carolina Gutierrez, Negar Asdaghi, Karlon Johnson, Gillian Gordon Perue, Erika Marulanda, Scott C. Brown, Dianne Foster, Tatjana Rundek

**Affiliations:** 1 Department of Neurology, University of Miami Miller School of Medicine, Miami, FL, United States; 2 Department of Public Health Sciences, University of Miami Miller School of Medicine, Miami, FL, United States; 3 American Heart Association, Dallas, TX, United States

**Keywords:** stroke, readmission, cardiovascular disease, epidemiology, rehospitalization, registry analysis

## Abstract

**Background:**

Hospital readmission is an important indicator of poor transition of care post-stroke. Data on characteristics of patients at highest risk for readmission is limited and necessary to inform effective interventions. The goal is to identify risk factors at hospital discharge that predict 30-day readmission in the Florida Stroke Registry (FSR).

**Methods:**

The study population included 45,877 patients discharged home or to rehabilitation with an ischemic stroke or intracerebral hemorrhage in the FSR between 2017 and 2019. The FSR is a voluntary statewide registry of stroke patients from 167 hospitals using data from Get With the Guideline-Stroke. Readmissions were ascertained by propensity matching FSR with the Florida Agency for Healthcare Administration dataset, which includes all hospital admissions in Florida. The primary outcome was 30-day hospital readmission for any cause, and secondary outcomes were vascular-related and stroke readmissions specifically. Multivariable logistic regression models identified patient characteristics that independently predicted 30-day readmissions, including sociodemographics, stroke clinical characteristics, in-hospital treatment, medical history, discharge status, and hospital characteristics.

**Results:**

A hospital readmission within 30 days was experienced in 12% of cases; 6% had a vascular-related readmission, and 3% a recurrent stroke. The following characteristics were independently associated with an increased risk of all-cause readmission: Medicare or Medicaid insurance, large artery atherosclerosis as the stroke mechanism, increased stroke severity, diabetes, atrial fibrillation, peripheral vascular disease, coronary artery disease, prior stroke, chronic renal insufficiency, and depression. The following characteristics were independently associated with a decreased risk of all-cause readmission: ambulation, treated dyslipidemia, tPA treatment, discharge mRS 0–2, and treatment at a comprehensive stroke center.

**Conclusions:**

The risk of 30-day hospital readmission was substantial, modifiable, and impacted by insurance status, medical history, stroke etiology and severity, stroke care, and functional status at discharge. These findings can inform strategies to target high-risk patients who can benefit from interventions to improve transitions of care post-stroke.

## Introduction

Readmission to the hospital is a common occurrence for stroke survivors, with approximately 25% of readmissions occurring within 1 month of the initial stroke (Bravata et al., 2007). In a previous publication using data from 16,952 Medicare fee-for-service beneficiaries discharged for ischemic stroke from hospitals in the Florida Stroke Registry from 2010 to 2013, we found that 15% of stroke patients were readmitted to the hospital for any cause within 30 days, with a median of 11 days between discharge from hospitalization for stroke and first hospital readmission (Gardener et al., 2019). Almost a quarter of these readmissions were due to an acute cerebrovascular event, and another 8% were due to pneumonia and urinary tract infections. In addition, racial and ethnic disparities were observed, with hospital readmissions more common among Black and Hispanic patients. These results were largely consistent with other national studies of hospital readmissions early post-stroke (Vahidy et al., 2017; Roberts et al., 2022). Hospital readmission is a key indicator of poor transition of care, as 30-day readmission after acute ischemic stroke increases mortality risk, as well as hospitalization expenses and length of stay in the hospital (Qiu et al., 2021). The economic burden of stroke readmissions on both the families of the patients and the healthcare system as a whole is noteworthy. Unplanned Medicare readmission was estimated in 2004 to be over $17 billion, and has only been increasing since then (Jencks et al., 2009). Therefore, decreasing readmission rates continues to be an objective of healthcare reform and transition of stroke care initiatives.

The frequent occurrence of hospital readmissions early after stroke, the high percentage of preventable causes for readmission, and the high economic burden of these readmissions highlight the need for improved post-discharge transition of care, the design of interventions to reduce hospital readmissions, and importantly, the identification of stroke survivors who are at the highest risk for readmission. In fact, the identification of high-risk patients is needed to inform future effective intervention strategies and studies. Data on the predictors of hospital readmission post-stroke is limited and not entirely consistent. Multiple studies have suggested an increased risk for readmission among older patients and those with more severe strokes, vascular comorbidities (e.g., diabetes, hypertension, coronary artery disease), and a prior stroke (Vahidy et al., 2017; Abreu et al., 2021; Loebel et al., 2022). The goal of the current study is to identify baseline risk factors after incident stroke as predictors for hospital readmission within 30 days post-stroke in the large state-wide Florida Stroke Registry.

We dedicate this manuscript to the memory of our Chair, colleague, mentor, and dear friend, Dr. Ralph L. Sacco. Dr. Sacco passed away in January 2023, after losing a battle with glioblastoma. Dr. Sacco founded the Florida Stroke Registry with a goal of improving stroke care and transition of care after stroke, and ultimately optimizing short-term and long-term outcomes for all stroke patients. His remarkable career as a stroke neurologist and clinical researcher-leader was dedicated to reducing disparities in stroke care. This manuscript represents Dr. Sacco’s mission to identify patients at highest risk for poor outcomes who can benefit most from interventions. We are grateful for his leadership, vision, and pioneering work that will continue in the Florida Stroke Registry to advance stroke care, stroke epidemiology, and brain health.

## Methods

### Data availability statement

The FSR uses data from Get With the Guideline-Stroke (GWTG-S). As GWTG-S is collected primarily for quality improvement, data-sharing agreements require an application process for other researchers to access data. Research proposals can be submitted at www.heart.org/qualityresearch and will be considered by the GWTG-S and the FSR Executive and Publication committees upon reasonable request.

### Florida Stroke Registry

In 2017, the Florida Stroke Registry (FSR) was established by Florida statute mandating participation of all Florida stroke centers. The FSR includes 167 out of 177 eligible hospitals. Data included in the registry is captured using the American Heart Association’s Get With the Guidelines–Stroke (AHA GWTG-S) quality improvement program. The current study includes stroke patients treated at FSR hospitals between 2017 and 2019. We restricted the study sample to patients who were discharged home or to rehab with an ischemic stroke or intracerebral hemorrhage (ICH).

The exposures of interest, captured in GWTG-S during the hospital evaluation for the index stroke event, are listed in Table 1 and include sociodemographics, clinical stroke characteristics, medical history, vascular risk factors, discharge status, in-hospital stroke care, and hospital characteristics.

The primary outcome of interest was all-cause hospital readmission within 30 days of discharge post-stroke. Readmission was ascertained by linking acute stroke data in the FSR with readmissions documented in the Florida Agency for Healthcare Administration (AHCA) dataset, which includes the dates and diagnosis codes for all hospital admissions in Florida. The AHCA dataset covered in 2017–2019 includes 8,592,450 unique admissions. Because social security numbers were only available in the AHCA dataset and no other unique identifiers were available, the FSR and AHCA datasets were propensity matched using the following variables: date of birth, discharge date, admission date, sex, race, ethnicity, zip code, age, and hospital ID. Out of 111,846 FSR stroke admissions, 92,672 were linked to the AHCA database, representing an 88% match, which is considered a highly successful matching. The dataset included 77,940 unique social security numbers, of which 76,533 were discharged before December 2019 and therefore at risk for a readmission within 30 days during the study period.

Secondary outcomes were vascular-related hospital readmissions, and readmissions due to any stroke. To identify the cause-specific hospital readmissions we utilized the ICD-10 principal diagnosis codes. Combined vascular readmissions included any stroke, TIA, atrial fibrillation, arrhythmia, coronary artery disease (CAD), heart disease, heart failure, myocardial infarction (MI), patent foramen ovale (PFO), heart valvular disease, atherosclerosis, carotid dissection, peripheral artery disease (PAD), pulmonary embolism (PE), stenosis/occlusion of cerebral arteries, coronary stent stenosis, and peripheral stent stenosis.

The University of Miami’s institutional review board approved this study.

### Statistical analysis

Out of the 76,533 patients in the FSR, 70,869 were admitted for an ischemic stroke or ICH as their index stroke (SAH and TIA as the index events were excluded), and of those 45,877 were discharged to home or to inpatient rehabilitation and were included in current analysis. We restricted the study to patients discharged home or to inpatient rehabilitation because we were interested in a patient population that was not under continuous medical care post-discharge as our ultimate goal with this work is to inform future intervention strategies in the community to improve transition of care post-stroke. First, we identified all patients who had at least one hospital readmission in the AHCA system at least 1 day after hospital discharge and less than 30 days after hospital discharge. Next, we identified those whose hospital readmissions included at least one for a vascular-related cause, and those whose hospital readmissions included at least one for stroke.

Multivariable logistic regression models were constructed to identify the independent predictors of (1) any hospital readmission within 30 days, (2) vascular-related hospital readmission within 30 days, and (3) stroke-related hospital readmission within 30 days. Due to the very large sample size available, all potential predictor variables were included simultaneously in the multivariable models. We examined the results of these models to confirm that multicollinearity between the predictor variables was not influencing conclusions. The missing indicator approach was used for variables with moderate to significant missingness, including NIHSS, discharge modified Rankin Scale (mRS), medical insurance, and ambulation at arrival and discharge. Prior exploratory analyses suggested that dyslipidemia treatment was associated with hospital readmission as well as dyslipidemia diagnosis, so this variable was trichotomized as no dyslipidemia, dyslipidemia treated with lipid-lowering medications, and untreated dyslipidemia. A two-sided *p*-value of 0.5 was used to determine statistical significance. All analyses were conducted using SAS version 9.4 (Cary, NC).

## Results

Out of 45,877 stroke patients, 12% experienced a hospital readmission within 30 days (*N* = 5,475), 6% experienced a vascular-related hospital readmission within 30 days (*N* = 2,557), and 3% experienced a hospital readmission for a recurrent stroke (*N* = 1,568). Among the first hospital readmissions within 30 days, 15.5% were due to pneumonia or other infections.

Table 1 shows the characteristics of the stroke survivors in the FSR overall as well as among those who had a hospital readmission for any cause within 30 days, those with a vascular-related readmission within 30 days, and those with a stroke readmission within 30 days. As shown, 46% were women, 64% non-Hispanic White, 21% non-Hispanic Black, and 15% Hispanic, with a mean age of 68 ± 14 years. In the study population, 91% had an ischemic stroke (9% ICH) and 75% were discharged home.

Table 2 shows the variables that are associated with the risk of all-cause hospital readmission, vascular-related hospital readmission, and stroke readmission within 30 days. The factors independently associated with an increased risk of all-cause readmission after mutual adjustment were Medicare or Medicaid insurance, large artery atherosclerosis as the stroke mechanism in the TOAST criteria (Adams et al., 1993), increased NIH stroke score (NIHSS), diabetes, atrial fibrillation, peripheral vascular disease, coronary artery disease, prior stroke, chronic renal insufficiency, and depression. The factors independently associated with a decreased risk of all-cause readmission after mutual adjustment were ambulation at discharge, treated dyslipidemia, tPA treatment, discharge mRS 0–2, and treatment at a comprehensive stroke center. For vascular-related readmissions, the factors that were independently predictive of an increased risk were having Medicare insurance, large artery atherosclerosis as the stroke mechanism, diabetes, atrial fibrillation, coronary artery disease, prior stroke, and carotid stenosis noted as pre-morbid risk factors in the GWTG-S data, while age over 90, Hispanic ethnicity, discharge mRS 0–2, and treatment at a comprehensive stroke center were independently associated with a decreased risk. For readmission with a primary diagnosis of any stroke, the factors that were independently predictive of an increased risk were large artery atherosclerosis as the stroke mechanism, diabetes, atrial fibrillation, coronary artery disease, and prior stroke, while increasing age, tPA, endovascular therapy, and treatment at a comprehensive stroke center were independently associated with a decreased risk. There was no significant difference in risk of readmission within 30 days between patients with an ischemic stroke vs. ICH as their index stroke event. Missingness for the NIHSS, mRS, and medical insurance did not predict the readmission outcomes.

## Discussion

The results from this large Florida state-wide stroke registry confirm that hospital readmission within the first 30 days post-stroke is common at 12%, as is readmission specifically for vascular causes (6%) and stroke (3%). The high frequency of readmissions after discharge from stroke hospitalization, particularly vascular- and stroke-related, highlights the need for novel interventions and hospital practices to be put in place for secondary prevention of stroke, especially for high-risk patients (e.g., those with large artery atherosclerosis as the stroke mechanism, diabetes, coronary artery disease, atrial fibrillation, and prior stroke). Our results demonstrated that the risk of hospital readmission for patients varied by medical history, stroke etiology and severity, stroke care, and functional status at discharge. A greater understanding of the risk factors for readmission is important to identify high-risk patients who can benefit from interventions to decrease the rate of future readmissions after stroke. In relation to the study population as a whole, because it included patients discharged directly home or to inpatient acute rehabilitation and excluded those discharged to nursing homes, hospice and subacute rehabilitation facilities, we would not expect them to be more likely than the general stroke patient population to have medical risk factors and comorbidities nor a higher risk of readmission.

Some of our results were consistent with the previous literature on readmissions after stroke, while others conflicted with the existing literature. A multicenter retrospective study conducted in China in 2021 found that older age, stroke severity, prior stroke, diabetes, indwelling urinary catheter, and admission to non-neurology floor during the initial admission were predictors for 30-day readmission among patients with ischemic stroke (Qiu et al., 2021). Similar findings were shown in a systematic review and meta-analysis that same year, which included 17 retrospective observational studies worldwide, and concluded that stroke history, diabetes, hypertension, atrial fibrillation, heart failure, and older age were risk factors for 30-day readmission (Deng et al., 2021). The results of that review and meta-analysis suggested that hyperlipidemia, coronary artery disease, smoking, and gender were not significant predictors of 30-day readmission. Like other studies, data from the Florida Stroke Registry showed that those with comorbidities such as diabetes and heart disease were at increased risk for 30-day readmission. Our data showed that patients with pre-existing coronary artery disease were at a significantly increased risk of readmission within 30 days, consistent with a recent retrospective case-control study (Loebel et al., 2022). Prior stroke was also found to be an important risk from our data and in the existing literature (Deng et al., 2021; Loebel et al., 2022). Dyslipidemia had been discussed as a risk factor in other papers (Deng et al., 2021); however, we found this comorbidity to be protective against readmission when it was treated. Previous studies have found that endovascular therapy was protective against readmission post-stroke (Vahidy et al., 2017), and we observed that it was associated with a decreased risk of stroke readmissions specifically. Unlike other papers that established older age as a risk factor for stroke readmission (Hirayama et al., 2018), we observed no association between age and all-cause readmission, and we observed that younger patients were more likely to have early readmission for a vascular cause, specifically a stroke. This was an unexpected observation and may be due to the competing risk of death. Further studies in the FSR will explore whether the incorporation of mortality data impacts this observed trend. We also observed no significant difference in the risk of 30-day readmission between patients with an index stroke that was ischemic vs. due to an ICH. A large population-based cohort study, providing national estimates of 30-day readmission between 2010 and 2015, showed that 30-day readmission was slightly higher in ICH patients (13.70%) compared to ischemic stroke patients (12.44%; Bambhroliya et al., 2018).

Several papers explored sex-differences in readmission risk, with some saying being female increased risk with others saying that there was no sex difference (Dennis et al., 2021). Our data did not find that sex was a significant predictor of readmission.

We observed a decreased risk of vascular-related readmissions among Hispanic patients compared to non-Hispanic White patients. The decreased risk of vascular readmissions among patients of Hispanic ethnicity in our study deserves further investigation. It is important to note that the observed decreased risk of vascular readmissions in Hispanic patients was independent of the other risk factors, including insurance status, stroke severity, medical history, and care in a Comprehensive Stroke Center. Future work in the Florida Stroke Registry will incorporate the racial and ethnic composition of the patient’s neighborhood, which may impact these findings. It is possible that the Hispanic patients may have more caregiver support and may be more likely to live in multi-generational households, and these factors may support transition of care post-stroke and offer protection against readmission. These hypotheses will be explored in future work among patients in the FSR, as our studies will examine the impact of living arrangements (i.e., who patients live with post-stroke) and their social support. In a cohort study of over 138,000 Medicare patients, Black patients and those who had more comorbidities and Medicare were more likely to have readmissions (Roberts et al., 2022). Unlike this data, we did not observe an increased risk of hospital readmission among Black patients.

Our results revealed several important and novel risk factors for readmission that can help identify high-risk patients. For example, mental health has been less explored in previous studies and our results showed that having depression was a significant risk factor for 30-day all-cause readmission but not for stroke or vascular-related readmissions specifically. This finding is meaningful, as post-stroke depression is a prevalent and biologically-plausible complication after stroke (Hackett and Pickles, 2014; Kutlubaev and Hackett, 2014; Wijeratne and Sales, 2021; Qiu et al., 2022). Further analysis of the role of depression in post-stroke readmissions would be both novel and warranted. Despite many comorbidities relating to cardiovascular health being explored in the past, there has been a lack of research on renal risk factors. We found that renal insufficiency also predicted an increased risk of all-cause readmission after stroke. Our results suggest that future work should explore whether post-stroke readmissions may be predicted using the Charlson Comorbidity Index (CCI), a weighted index that combines both disease numbers and severity and includes renal disease. Recently, the CCI was shown to be useful in estimating the readmission rate within 28 days, 3 months, and 6 months among patients with heart failure (Wei et al., 2023). Lastly, data has also been limited on ambulation status among stroke survivors, and our results suggested that patients who were not ambulating independently at discharge had an increased risk of hospital readmission.

The results also provide valuable evidence showing that being treated for a stroke at a certified comprehensive stroke center is associated with a reduced risk of readmission for all causes, vascular causes, and stroke specifically. In 2004 the Florida Stroke Act was signed into law, which set criteria for Comprehensive Stroke Centers to provide the best evidence-based care to stroke patients (Marulanda et al., 2023). The purpose was to improve stroke patient access to advanced diagnosis and treatment which has evolved over the past two decades including tPA, endovascular therapy, and enhanced neuroimaging. The current results confirm the hypothesis that the guideline-driven standardized metrics required for comprehensive stroke center certification improve patient outcomes a month after discharge. Previous data have also suggested that treatment at a Comprehensive Stroke Center improves survival in-hospital (Iihara et al., 2014). Insurance status can also be an important predictor of readmission as it is a proxy measure for socioeconomic status, and therefore related to a patient’s ability to adhere to prescribed medications, attend medical appointments and rehabilitation, and seek preventive healthcare. Medical insurance status relates to the patient’s ability to afford medical care and therefore their willingness to seek necessary medical care post-stroke.

Important strengths of our study include a large sample size which has been linked to statewide data resulting in a comprehensive data collection of patient characteristics and care, allowing for the identification of independent risk and protective factors for readmission. The most noteworthy limitation of the current study is the lack of data on mortality within the first 30 days post discharge, which may have introduced some bias in relation to variables associated with a high risk of early mortality post-stroke (e.g., increased age). In addition, we did not include in our outcome emergency room visits without hospital readmission, and this is also a key indicator of poor transition of care post-stroke. Future studies in this state-wide stroke registry will examine the predictors of more long-term readmissions (90-day and one-year); incorporate both ER visits and mortality; explore social determinants of health, neighborhood-level characteristics, and post-discharge health behaviors, in an effort to identify patients and barriers to care more comprehensively. Ultimately these data will be used to create a prediction index for post-stroke hospital readmissions so that we may identify patients and communities at highest risk for poor transition of care and incorporate appropriate interventions for these patients, communities, and social determinants of health to decrease readmission rates post stroke. The current study examined readmissions for any type of stroke that occurred within 30 days post-discharge after an initial ischemic stroke or ICH in the FSR, and future studies will look at the risk of 30-day readmission for specific stroke subtypes.

These results emphasize and are bolstered by previous work in the Florida Stroke Registry indicating that a large portion of hospital readmissions are due to preventable causes, including pneumonia, other infections, and vascular events. These observations validate our continued efforts in the Florida Stroke Registry to improve transitions of care practices and identify those patients who are at highest risk for preventable poor outcomes.

## Figures and Tables

**TABLE 1:** Exposures of interest captured in GWTG-S during hospital evaluation for the index stroke event.

	Study population (*N* = 45,877)	All cause readmission (*N* = 5,475)	Vascular readmission (*N* = 2,557)	Stroke readmission* (*N* = 1,568)
Age <50, %	10	9	9	11
Age 50–59, %	17	15	16	18
Age 60–69, %	25	23	23	22
Age 70–79, %	26	27	29	27
Age 80–89, %	18	20	19	18
Age 90+, %	5	6	5	4
Male, %	54	54	55	55
Black, %	21	21	21	23
Hispanic, %	15	14	13	14
White, %	64	65	66	63
Private insurance, %	32	30	30	31
Medicare, %	31	34	33	32
Medicaid, %	4	5	4	4
Self/no insurance, %	6	5	5	6
Unknown insurance, %	28	27	28	27
Stroke mechanism: large artery atherosclerosis, %	10	12	15	13
NIHSS 0	14	13	15	16
NIHSS 1–4	36	32	35	35
NIHSS 5–15	20	22	20	19
NIHSS 16+	5	6	6	5
NIHSS missing	25	27	25	26
Ambulation before and after hospitalization, %	55	48	54	53
Ambulation before hospitalization only, %	17	21	19	19
Not ambulatory before hospitalization, %	2	4	2	2
No ambulation at discharge, %	9	11	9	10
Ambulation unknown before and after hospitalization, %	16	17	16	15
Diabetes, %	29	33	33	34
Hypertension, %	69	72	72	71
Treated dyslipidemia, %	81	76	81	80
Untreated dyslipidemia, %	1	2	1	2
No dyslipidemia, %	18	22	17	18
Atrial fibrillation, %	16	21	21	17
Peripheral vascular disease, %	3	5	5	4
Coronary artery disease, %	19	24	25	22
Previous stroke, %	22	25	25	27
Carotid stenosis, %	3	4	4	3
Chronic renal insufficiency, %	6	10	8	7
Depression, %	8	9	8	8
Smoker, %	18	17	17	17
Drug/alcohol use, %	6	6	6	6
Overweight/obese, %	23	22	22	23
DVT/PE, %	1	1	1	1
Index stroke: intracerebral hemorrhage, %	9	11	8	10
Index stroke: ischemic stroke, no tPA treatment, %	76	77	78	79
Index stroke: ischemic stroke, with tPA treatment, %	15	13	13	12
Endovascular therapy, %	6	6	6	4
mRS 0–2 at discharge, %	37	30	34	34
mRS 3+ at discharge, %	28	34	30	31
mRS missing, %	35	36	36	35
Discharged home, %	75	69	73	74
Comprehensive Stroke Center, %	41	40	40	40
Primary Stroke Center, %	49	48	48	48
Other center type, %	11	12	12	12
Teaching hospital, %	29	29	29	30

* Includes all stroke types.

**TABLE 2:** Risk factors for all-cause hospital readmission, vascular-related hospital readmission, and stroke readmission within 30 days.

	OR* for all cause readmission	95% confidence interval	OR* for vascular readmission	95% confidence interval	OR* for stroke readmission	95% confidence interval
Age 50–59 vs. <50	1.01	0.89–1.15	0.99	0.84–1.18	0.92	0.75–1.13
Age 60–69 vs. <50	0.98	0.87–1.10	0.87	0.73–1.03	**0.75**	**0.62–0.92**
Age 70–79 vs. <50	0.98	0.87–1.11	0.97	0.82–1.15	0.84	0.68–1.03
Age 80–89 vs. <50	1.01	0.88–1.16	0.86	0.71–1.03	**0.76**	**0.61–0.95**
Age 90+ vs. <50	1.03	0.87–1.22	**0.74**	**0.58–0.96**	**0.59**	**0.43–0.82**
Male	0.97	0.91–1.03	1.01	0.93–1.10	0.99	0.89–1.10
Black vs. White	0.98	0.91–1.07	1.00	0.89–1.12	1.03	0.90–1.18
Hispanic vs. White	0.94	0.86–1.03	**0.84**	**0.74–0.96**	0.89	0.76–1.04
Medicare vs. Private	**1.15**	**1.07–1.24**	**1.13**	**1.02–1.26**	1.08	0.94–1.24
Medicaid vs. Private	**1.31**	**1.13–1.53**	1.08	0.86–1.35	1.06	0.81–1.39
Stroke mechanism: large artery atherosclerosis	**1.28**	**1.17–1.41**	**1.69**	**1.50–1.90**	**1.40**	**1.19–1.64**
NIHSS 0 vs. 1–4	1.03	0.94–1.14	1.12	0.98–1.27	1.14	0.98–1.14
NIHSS 5–15 vs. 1–4	**1.36**	**1.18–1.57**	1.22	0.99–1.49	1.10	0.83–1.57
NIHSS 16+ vs. 1–4	**1.18**	**1.09–1.29**	1.05	0.93–1.19	1.06	0.91–1.29
NIHSS missingvs. 1–4	1.10	1.00–1.21	1.08	0.95–1.23	1.04	0.89–1.21
Ambulation before only vs. before and after	1.09	0.98–1.20	1.01	0.88–1.17	1.12	0.94–1.33
Ambulation never vs. before and after	**1.44**	**1.21–1.70**	0.90	0.68–1.19	0.89	0.62–1.28
Ambulation not at dc vs. before and after	**1.26**	**1.13–1.40**	1.08	0.92–1.27	1.19	0.97–1.45
Ambulation unknown vs. before and after	**1.21**	**1.10–1.34**	1.10	0.95–1.27	1.08	0.90–1.29
Diabetes	**1.19**	**1.11–1.27**	**1.16**	**1.05–1.27**	**1.18**	**1.05–1.33**
Hypertension	1.02	0.94–1.09	1.06	0.96–1.18	1.07	0.93–1.21
No dyslipidemia vs. treated dyslipidemia	**1.33**	**1.21–1.49**	0.98	0.84–1.15	0.97	0.79–1.19
Untreated dyslipidemia vs. treated	**1.37**	**1.20–1.86**	1.00	0.71–1.42	1.11	0.73–1.70
Atrial fibrillation	**1.32**	**1.20–1.41**	**1.48**	**1.33–1.65**	**1.16**	**1.01–1.34**
Peripheral vascular disease	**1.25**	**1.08–1.44**	1.17	0.96–1.44	1.21	0.93–1.57
Coronary artery disease	**1.29**	**1.20–1.39**	**1.31**	**1.18–1.45**	**1.16**	**1.01–1.32**
Previous stroke	**1.11**	**1.04–1.19**	**1.12**	**1.02–1.24**	**1.29**	**1.14–1.46**
Carotid stenosis	1.13	0.95–1.33	**1.25**	**1.01–1.56**	1.14	0.85–1.53
Chronic renal insufficiency	**1.37**	**1.23–1.52**	1.13	0.96–1.31	0.97	0.79–1.19
Depression	**1.13**	**1.02–1.25**	1.00	0.86–1.16	0.93	0.77–1.12
Smoker	1.01	0.93–1.09	0.98	0.87–1.10	0.90	0.78–1.04
Drug/alcohol use	1.08	0.95–1.22	0.93	0.77–1.11	1.00	0.80–1.25
Overweight/obese	0.93	0.86–1.00	0.95	0.86–1.05	0.95	0.83–1.08
DVT/PE	1.13	0.86–1.47	1.03	0.70–1.52	0.92	0.55–1.54
Index stroke: ICH vs. ischemic without tPA	0.96	0.84–1.11	1.02	0.82–1.27	1.15	0.87–1.51
Index stroke: ischemic with tPA vs. without tPA	**0.80**	**0.73–0.88**	0.89	0.78–1.01	**0.78**	**0.65–0.92**
Endovascular therapy	0.94	0.82–1.08	0.91	0.75–1.11	**0.72**	**0.55–0.95**
mRS 0–2 vs. 3+	**0.79**	**0.72–0.87**	**0.87**	**0.77–0.99**	0.91	0.78–1.08
mRS missing vs. 3+	0.94	0.86–1.03	0.93	0.82–1.05	0.96	0.81–1.12
Discharged home	0.93	0.86–1.00	1.06	0.95–1.18	1.07	0.93–1.23
Teaching hospital	1.06	0.98–1.13	1.05	0.95–1.15	1.10	0.97–1.24
PSC vs. CSC	0.99	0.93–1.06	1.00	0.91–1.10	0.98	0.87–1.10
Other center type vs. CSC	**1.18**	**1.07–1.30**	**1.15**	**1.00–1.32**	**1.22**	**1.03–1.45**

* All variables in this table are mutually adjusted. Bold values indicate statistical significance, *p* < 0.05.

## Data Availability

The datasets presented in this article are not readily available because the FSR uses data from Get With the Guideline-Stroke (GWTG-S). As GWTG-S is collected primarily for quality improvement, data-sharing agreements require an application process for other researchers to access data. Research proposals can be submitted at www.heart.org/qualityresearch and will be considered by the GWTG-S and the FSR Executive and Publication committees upon reasonable request. Requests to access the datasets should be directed to www.heart.org/qualityresearch.
